# The commercial roots of the genomic commons

**DOI:** 10.1177/03063127241310122

**Published:** 2025-01-18

**Authors:** Steve Sturdy

**Affiliations:** Science, Technology and Innovation Studies, The University of Edinburgh, Edinburgh, Scotland, UK

**Keywords:** intellectual property, commons, biotechnology, genomics, innovation systems

## Abstract

Accounts of the origins of the genomic commons typically focus on the development of public repositories and data-sharing agreements. This article tells a different story. During the 1990s in the United States, efforts of private companies to prevent the patenting of certain kinds of DNA sequences were essentially a conservative response to shifts in the sociotechnical constitution of the pharmaceutical innovation system, and to the operation of intellectual property as one of the key knowledge control regimes that regulate that system. In this context, the idea of ‘the commons’ was rehabilitated from earlier tragic theorizations to argue that industry’s ability to deliver new pharmaceutical products would be better served if certain kinds of intellectual property were left in the public domain. The genomic commons is not a neutral space of disinterested scientific research that naturally aligns with some abstract ‘public good’, but is part of an innovation system that has evolved to serve the interests of a range of stakeholders, among which the big pharmaceutical companies enjoy a dominant position.

Talk of a genomic or other kind of data ‘commons’ comes freighted with connotations of public virtue. The rapid release of genomic data is not only ‘necessary for the advancement of scientific research and the public good’, but also suggests ‘a reconceptualization of genomic data as a public good’ in its own right, write scholars Contreras and Knoppers (2018, pp. 430, 438) in a review of the genomic commons. They go on to set public virtues against the ‘threat of propertization of the genome’ and the potentially inhibitory effects of private ownership of genomic data (Contreras & Knoppers, 2018, pp. 442–446). As Prainsack (2019, p. 3) notes, the frequent repetition and wide circulation of such tropes have ‘made the commons a symbol of opposition to “private property” and commercial interest’. This agonistic reading is reinforced by a historical narrative of the origins of the genomic commons which pits the efforts of academic scientists and public and charitable research funders against the proprietary claims of commercial organizations such as Celera Genomics (Contreras & Knoppers, 2018, p. 443), and which foregrounds the growth of the public repositories used to collate data from the Human Genome Project (HGP) and the data-sharing agreements which have helped to populate them (Contreras & Knoppers, 2018, p. 430 et passim; also e.g., Maxson Jones et al., 2018). Here too, the virtues of publicness are given a central role: It was the ‘sense of public stewardship’ exhibited by the HGP’s scientific leaders ‘that contributed to the community-spirited character of several HGP policies’, while the resulting genomic commons itself represents a ‘public spirit’ (Contreras & Knoppers, 2018, p. 434) that is directly antithetical to private and commercial self-interest.

I tell a rather different origin story. Rather than privileging public-sector actors, I foreground the role that private companies played in preventing the patenting of certain kinds of DNA sequences in the United States. They did so not out of any public spiritedness, but to shore up the influence they enjoyed within the larger pharmaceutical innovation system. In this context, the idea of ‘the commons’ was rehabilitated from earlier tragic theorizations to argue that industry’s ability to deliver new pharmaceutical products would be better served if certain kinds of intellectual property were left in the public domain. The genomic commons is not a neutral space of disinterested scientific research that naturally aligns with some abstract ‘public good’, but is part of an innovation system that has evolved to serve the interests of a range of stakeholders, among which the big pharmaceutical companies enjoy a dominant position. Uncritical association of the commons with public virtue obscures the extent to which it serves and is a product of private interests. In so doing, it inhibits more searching reflection on what kind of biomedical innovation system might best serve the public good.

Empirically, this article is based on a wide range of published documents, mostly from the period 1990-2000, including research articles, official publications, news reports, patents, court judgements, and published interview transcripts with key players, which were analyzed to reconstruct and contextualize arguments and activities relating to the patentability of DNA sequences during that period. Theoretically, I develop Hilgartner’s (2017, p. 10) insight that patent law can be regarded as one among many ‘knowledge-control regimes’ that regulate the distribution and use of the results of scientific research. Hilgartner’s own research has focused primarily on public genome research, and on the mostly voluntary knowledge-control regimes that operate there. Here I extend that project to look at intellectual property claims and counterclaims as they relate to the products of genomic research. In so doing, I not only confirm and show how intellectual property law operates as a knowledge-control regime; I also show how, in the case of genomic research, that regime spanned both public research institutions and commercial organizations, as well as regulatory agencies and legal authorities.

This article is also informed by anthropological perspectives on property. Starting from the idea that ‘property is ultimately a relationship between people in relation to “things”’ (Macfarlane, 1998, p. 112), I respond to anthropologists’ challenge ‘to attend to significant social relations co-produced through IP regulation’ (Coombe & Chapman, 2020, p. 5; also Strathern, 2005). I show how decisions about what kinds of genomic ‘things’ might be claimed as intellectual property served to determine what kinds of relationships were possible between different sorts of ‘people’. The development of intellectual property law, including determinations about what may *not* be treated as legal property, has served to structure the pharmaceutical innovation system by prescribing and delimiting what rights and responsibilities pertain to different actors within that system, and hence the kinds of relationships they can forge with one another and with the products of genomic research.

## Intellectual property, medicine and the public good to 1980

Ownership of intellectual property has been pivotal to the development of the US pharmaceutical innovation system since the mid-19th century. The principal rationale for awarding patent rights is to benefit the public by incentivizing the development of useful new inventions. But just what kinds of things should be patented, who should own them, and what rights ownership should entail, have all been subject to contestation and revision (Dutfield, 2020; Gabriel, 2014). Many countries with well-developed pharmaceutical sectors resisted patenting medicinal products until well into the 20th century, for fear that people with serious health problems were peculiarly vulnerable to exploitation by companies holding monopolies on sought-after treatments (e.g., Cassier, 2008; Gaudillière, 2008). The United States was much more inclined to favor incentives for private innovation over public protection, and led the way in allowing claims on an increasingly wide range of medicinal products. In Parthasarathy’s (2017, p. 22) succinct summary, in the US, ‘The inventor’s interest … was the public interest’.

This balance of interests was decisively reasserted during the 1960s and 1970s. As the US pharmaceutical industry expanded rapidly in the early post-War years, driven by its success in launching new products and boosted by a favorable patent regime, some worried that companies were putting profit before patients, charging excessive prices for products that were not always effective or safe. In 1961, Senator Estes Kefauver introduced draft legislation to curtail such practices, including reducing the period of patent exclusivity on pharmaceutical products. His timing was inauspicious. Amid the anxieties of the Cold War, the pharmaceutical industry was widely celebrated as an American success story, symbolizing the innovative energy and productivity of capitalist enterprise (Tobbell, 2012). With lobbyists and legislators warning that Kefauver’s proposals would shackle the industry, all mentions of patent restrictions were stripped from his bill (Gabriel, 2016, pp. 592–598; Parthasarathy, 2017, pp. 34–38).

An emboldened pharmaceutical industry went on the offensive, seeking even greater freedom to exploit patents than it already enjoyed. Its targets were the universities and government agencies. Public-sector researchers were generally not averse to commercialization of their discoveries. But they preferred to leave that to industry, while cultivating for themselves an identity as commercially disinterested, public-minded scientists. Consequently, insofar as they sometimes sought to patent the results of their research, it was more often to prevent predatory commercial practices than to boost their own or their institutions’ income: Patent ownership enabled a measure of control over how public-sector research was commercialized, including preventing monopolies by awarding non-exclusive licenses, and ensuring that production met agreed standards of quality (Cassier & Sinding, 2008; Eisenberg, 1996, pp. 1671–1677; Metlay, 2006, pp. 568–581). This approach was embraced in the immediate post-war years by the National Institutes of Health (NIH), which by the late 1950s was sponsoring the vast majority of government-funded medical research in the US. The NIH encouraged its researchers to patent their research, but retained title on those patents to ensure that they were commercialized on a competitive, non-exclusive basis (Berman, 2008, p. 843; Eisenberg, 1996, pp. 1675–1676). In effect, NIH cast itself in a stewardship role, holding patents to prevent the products of publicly funded science from becoming private monopolies.

Pharmaceutical companies challenged that role, including adopting what NIH’s patent counsel later described as ‘a virtual boycott’ of NIH-held patents (Government Patent Policy, 1976, p. 723). By the early 1970s, the NIH had softened its policy considerably: Provided universities implemented what the agency considered appropriate patent management policies, the NIH now authorized them to retain title on patents and manage them as they saw fit, including granting companies exclusive licenses (Eisenberg, 1996, pp. 1677–1685; Yi, 2015, pp. 141–146). By the mid-1970s, a broad coalition of university leaders, industrialists, politicians, and Chicago School economists was calling on Congress to overhaul its intellectual property policies in order to facilitate the commercialization of publicly-funded research (Berman, 2008, pp. 849–858; Nik-Khah, 2014). As Cold War triumphalism gave way to consternation at the rapid emergence and innovative vigor of German and Japanese competitors, in 1980 Congress passed the Bayh-Dole Act, freeing universities to patent and license discoveries arising from federally funded research as they wished,^1^ while the less well-known Stephenson-Wydler Act, passed two months earlier, made it a requirement for federal laboratories to pursue technology transfer and commercialization (Berman, 2008, pp. 849–858; Yi, 2015, pp. 158–166).

These measures effectively turned the NIH’s public stewardship role on its head. Previously the agency had sought and held patents with the aim of protecting the public from the more harmful consequences of commercialization, principally by preventing monopolies. Now, its public responsibilities were statutorily redefined to include actively enabling commercialization, including exclusive licensing of the research it funded. Stephenson-Wydler and Bayh-Dole thus effected a major adjustment to the way that intellectual property operated within the US pharmaceutical innovation system, both as a knowledge control regime and as a nexus in the relationships between different kinds of people—funders, researchers, and commercial companies—who participated in that system.

## Patenting and the birth of biotech

The birth and development of the biotechnology industry was intimately bound up with these transformations in the US patent regime. In 1974, molecular biologists Stanley Cohen and Herbert Boyer, based at Stanford and the University of California at San Francisco respectively, announced a novel method of introducing foreign DNA sequences into micro-organisms, and inducing them to produce the proteins those sequences coded for. At the urging of Niels Reimers, Director of Technology Licensing at Stanford and a leading figure in the new cadre of university patent managers, Cohen and Boyer applied for patents on their method and on any recombinant organisms it might be used to create (Hughes, 2011, pp. 20–22; Yi, 2015, pp. 150–153). Start-up companies were quickly set up to develop the new recombinant DNA technology with a view to manufacturing drugs such as insulin and interferon. These included Genentech, founded in 1976 by Boyer and venture capitalist Robert Swanson (Hughes, 2011, pp. 29–48), and Biogen, established two years later in a similar partnership between molecular geneticists and venture capital (Rasmussen, 2014, pp. 106–107). Meanwhile, pharmaceutical companies channelled funding into academic laboratories to support product-oriented recombinant DNA research (Kenney, 1988).

The prospects for these new commercial ventures remained uncertain, however. Much depended on whether Cohen and Boyer’s patents would be granted. And this in turn depended on the outcome of an earlier patent application, filed in 1972 by Ananda Chakrabarty, a researcher at General Electric, for an oil-digesting bacterium created using an earlier approach to genetic modification (Kevles, 1994). Chakrabarty’s method was far less powerful than Cohen and Boyer’s recombinant DNA technology, but it set an important precedent: If approved, Chakrabarty’s patent would establish that modified living organisms were patentable subject matter. Brought to public attention by the media reports of Cohen and Boyer’s achievement, this quickly became a matter of fierce controversy. Objections to the Chakrabarty and Cohen-Boyer claims covered a wide range of potential public harms, from the moral harms that could result from patenting life-forms, to the social, ethical and environmental risks associated with engineering living organisms, to fears that patenting would inhibit the circulation of scientific findings. Patent advocates responded with the same arguments as they were deploying in the debate over patenting publicly funded research—that commercial development of biotechnology would benefit the public by opening up new lines of pharmaceutical innovation, bringing new medicines to market and driving economic growth and competitiveness (Berman, 2012, pp. 58–81; Kevles, 1994, pp. 121–132; Yi, 2015). With Boyer himself urging that ‘the technology gets transferred to private industry so that public benefits come out as soon as possible’ (quoted in Hughes, 2011, p. 72), and amicus briefs from the pharmaceutical industry pushing the same line, in June 1980 the US Supreme Court ruled that patent law did not exclude claims on living organisms, and Chakrabarty’s patent was therefore admissible (Kevles, 1994). The judgement cleared the way for the US Patent and Trademark Office (PTO) to approve the Cohen and Boyer patents, beginning in December 1980 with their process patent, followed by their product claims on recombinant prokaryotic and eukaryotic organisms in 1984 and 1986 respectively (Hughes, 2001; Yi, 2015, pp. 272, n. 146).

Along with the Bayh-Dole Act, the Chakrabarty and Cohen-Boyer patent decisions created the conditions for the establishment of a new field of commercial enterprise. Patents were central to the biotech business model. University-based researchers possessed the technical ability to create recombinant organisms of use in producing commercially valuable proteins, but they generally lacked the resources to undertake commercial production. Instead, they patented their discoveries, and licensed them to larger companies to develop and market. Patents, rather than products, were thus at the heart of the biotech business model, serving as indicators of commercial promise that could be leveraged to secure further investment in the short term, and as assets that could be licensed for longer-term income (Doganova & Muniesa, 2015; Fortun, 2008). The production and movement of patents also had the effect of blurring the boundary between academic and commercial spaces within the larger pharmaceutical innovation system. Before the 1970s, university researchers had tended to maintain an arms-length relationship to any commercial activities arising from their findings, often by delegating them to non-profit organizations such as the Research Corporation (Apple, 1989; Mowery & Sampat, 2001). From the mid-1970s, in contrast, growing numbers of molecular geneticists set up their own biotechnology companies, solicited commercial funding, and took an active role in managing their intellectual property, while retaining their university positions and continuing to receive public research funding (Kenney, 1988; Rasmussen, 2014). By 1984, as much as a quarter of all biotechnology research in American universities was supported by industry, while nearly a half of all biotechnology companies funded academic research (Blumenthal et al., 1986).

Approval of the Chakrabarty and Cohen-Boyer patents also paved the way for patents on whole new classes of things. The first wave of biotech patents involved recombinant organisms engineered to produce medical and other proteins. A distinctive feature of these patents was their inclusion of DNA sequence information, as part of the specification of the organism being claimed and the protein it was designed to produce. DNA sequencing was still a slow and painstaking process, but it was invaluable as a means of specifying complex biological products, particularly vaccines, which previously had proved difficult or impossible to describe with the degree of precision and specificity necessary to satisfy the requirements for patenting (Huzair & Sturdy, 2017; see also Cassier, 2008; Gaudillière, 2008; Kevles, 2007; Pottage & Sherman, 2010). Over the course of the 1980s, DNA sequences also began to be claimed as patentable objects in their own right. By the mid-1980s, researchers had succeeded in mapping DNA marker sequences linked to human diseases including Duchenne muscular dystrophy and cystic fibrosis (Caskey, 1987; Cooper & Schmidtke, 1986), and now filed patents claiming those sequences as diagnostic tools (e.g., Barker et al., 1986; see also Joyce, 1987). By the end of the decade, as scientists located and sequenced the Duchenne and cystic fibrosis genes themselves, they also claimed patents on those genes (Chandrasekharan et al., 2010; Kunkel et al., 1993). The proliferation of DNA patents was in turn encouraged and enabled by the patent authorities. The tone was set by the Court of Appeal in the Federal Circuit, set up in 1982 specifically to adjudicate in patent disputes, which applied standards of patentability carried over from pharmaceutical innovation, including a low non-obviousness threshold that effectively opened the door to routine discovery and patenting of nucleotide sequences (Burk & Lemley, 2002; Rasmussen, 2014).

Criticism of these new arrangements was mostly muted or marginal. Moral, ethical, and occasionally scientific concerns about patenting genetic material rumbled on in the background. But as long as biotechnological research remained scientifically as well as commercially fruitful, molecular geneticists generally accepted the convergence of public and commercial interests in their laboratories and their work, while biotech patents were incorporated into the network of knowledge control regimes that mediated the pharmaceutical innovation system more generally. That complacency would not last, however. The opportunities for technical and commercial innovation presented by the new molecular biotechnology were still being explored and extended, and would result, in the early 1990s, in an eruption of novel patent claims that threatened to destabilize key relationships within the pharmaceutical innovation system.

## The trouble with ESTs

An important technique in recombinant DNA biotechnology is the production of so-called complementary DNA (cDNA). Generated by reverse transcription of messenger RNAs extracted from cells in which protein synthesis is taking place, cDNAs effectively reproduce the parts of the genome that code for those proteins. Introduced into yeasts or bacteria, cDNAs make it possible to manufacture the relevant proteins. cDNA sequences therefore feature prominently in the specification of recombinant DNA patents. But cDNAs can also be used in a more exploratory fashion, to investigate what genes were being expressed in particular tissues. By the late 1980s, cDNAs extracted from a wide range of species and tissues were accumulating in cDNA libraries. Given the limitations of the sequencing techniques available at that time, however, cDNAs were being added to libraries much faster than they could be sequenced or otherwise characterized.

In 1991, biologist Craig Venter—at that time employed at the National Institute of Neurological Disorders and Stroke, one of the National Institutes of Health—devised a method of uniquely identifying different cDNAs by sequencing short sections of between 300 and 500 nucleotides. Venter called these identifying sequences ‘expressed sequence tags’ or ESTs. Using newly available automatic DNA sequencers, he was able to generate ESTs at the rate of almost 100 per day (Adams et al., 1991, 1992). ESTs were of immediate value for cataloguing and managing cDNA libraries. But they also had potential commercial value as means of identifying and testing for genes that might be associated with disease. Alerted to this by Max Hensley, a patent attorney at Genentech, and mindful of NIH’s statutory duty to promote commercialization of the research it funded, Reid Adler, the director of NIH’s Office of Technology Transfer, persuaded Venter to file patent applications on his ESTs, claiming not just the tag sequences, but also the larger, as yet unknown cDNA and gene sequences that the ESTs identified. Venter filed his first application in June 1991, claiming 337 sequences derived from human brain tissue (Anderson, 1991; Andrews, 1991; Roberts, 1991a); a second application, claiming a further 2375 ESTs and their associated genes, followed in February 1992 (Roberts, 1992a). Five months later, Venter left the NIH to direct a new organization, the Institute of Genomic Research (TIGR), funded with $70 million in venture capital, with the rights to any patents it secured to be assigned to a for-profit partner company called Human Genome Sciences (Kolata, 1992). Two other startup companies—Incyte and Millennium Pharmaceuticals, established in 1991 and 1993 respectively—also began identifying and patenting ESTs on an increasingly large scale. By early 1994, Human Genome Sciences had filed patents on 9,900 sequences, while Incyte claimed to have filed for 40,000 more (Anderson, 1993, 1994; also Millennium Pharmaceuticals, Inc., 1996).

These developments provoked consternation among scientists. Human genome researchers, in particular, condemned the NIH’s patent applications, arguing that the patents could ‘impede the open exchange of information’ between scientists (Roberts, 1991a, p. 184; also Andrews, 1991; Roberts, 1992a). Their objections, they stressed, were not to patenting DNA sequences in general, which they maintained was vital for bringing the benefits of biomedical research to the public, but specifically to patenting ESTs. At issue was how Venter’s method of generating ESTs disrupted the existing economy of DNA sequence data. DNA sequencing, patenting, and commercialization usually proceeded piecemeal, as particular sequences were shown to have specific scientific, medical or industrial uses (Hilgartner, 2017, pp. 63–122; Knorr Cetina, 1999). In contrast, Venter’s systematic mining and sequencing of cDNA libraries looked more like industrial mass production; while wholesale patenting of ESTs threatened to concentrate ownership of large parts of the human genome in a small number of organizations. As the Office of Technology Assessment observed, the ‘orderly process’ of identifying and patenting genes ‘was altered’ by Venter’s move to mass production and mass patenting (US Congress, Office of Technology Assessment, 1994, pp. 1–42). Hence the sense of outrage among molecular biologists: Venter’s approach, in James Watson’s scathing assessment, was so mechanical that ‘virtually any monkey’ could do it, while others feared that treating ESTs as intellectual property would result in ‘a mad scramble for patents’ (Roberts, 1991a, p. 184).

Scientists’ concerns were shared by many in industry. A number of commercial organizations, including the Pharmaceutical Manufacturers Association and the Industrial Biotechnology Association, objected to the NIH’s move to patent ESTs, while the biotech sector as a whole was reportedly ‘deeply divided’ over the issue (Roberts, 1992a, 1992b). Biotechnology and pharmaceutical companies were linked into the same networks of DNA circulation as academic laboratories, and they too feared that mass production and bulk ownership of DNA sequences would destabilize existing relationships and systems of exchange. Pharmaceutical companies, in particular, feared that the proliferation and accumulation of intellectual property in DNA data ‘will merely add to the thicket of patent rights that firms must negotiate their way past before they can get products on the market’ (Eisenberg, 1992, p. 904; also Kolata, 1992; Yi, 2015, p. 166).

Pharmaceutical companies responded to this threat in different ways. One strategy was to buy into the mass production of gene patents through wholesale licensing deals. In May 1993, SmithKline-Beecham struck a $125 million deal to secure exclusive access to Human Genome Sciences’ collection of ESTs. Ten months later, Hoffman-LaRoche invested $70 million in a collaboration with Millennium Pharmaceuticals, aimed at identifying genes associated with diabetes and obesity (Bylinsky, 1994; Fisher, 1994). Merck chose a radically different solution. In September 1994, the company announced the launch of the Merck Gene Index Project (MGIP), channeling an initial $10 million of funding to researchers at Washington University and elsewhere to ‘stimulate a great increase in the rate of public sequencing of expressed sequences’—isolating cDNAs, tagging them with ESTs, and releasing them to public repositories such as GenBank (Merck & Co., Inc., 1995; Williamson, 1999; Williamson & Elliston, 1994). With this support, academic researchers were able to produce and publish ESTs at a rate that rivalled anything being achieved in the private sector at that time, pre-empting the appropriation of ESTs and instead dedicating them to the public domain.

Meanwhile, scientific and commercial excitement was coming to focus on another kind of DNA sequence data. Researchers were increasingly optimistic that information about genetic variation at the level of single nucleotide polymorphisms (SNPs) would soon give rise to a new generation of diagnostic and therapeutic technologies. Identification and ownership of SNPs accordingly became the next site of commercial interest in DNA sequences. In July 1997, diagnostics giant Abbot Laboratories announced its intention to invest up to $45 million in the Paris-based biotech company Genset to build two proprietary SNP databases, and to file patents on SNPs of potential commercial interest; while a consortium including Bristol-Myers Squibb, Millennium Pharmaceuticals, and the DNA diagnostics company Affymetrix was reported to be preparing a similar initiative (Marshall, 1997). Like the earlier furore around ESTs, these initiatives provoked fears that ownership and control of large amounts of DNA sequence data would become concentrated in a few commercial enterprises.

This time, efforts to head off the threat of enclosure included strong public-sector involvement. The National Advisory Council for Human Genome Research (NACHGR) had been established in 1990 to advise Government and the NIH on issues connected to the Human Genome Project. At its meeting of 11 September 1997, NACHGR turned its attention to the threatened privatization of SNPs. Following a discussion led by Alan Williamson—Vice President for Research Strategy at Merck, chief architect of the Merck Gene Index Project, and recent appointee to NACHGR—the Council resolved to launch what one observer called a ‘pre-emptive strike’ by creating a public repository of human SNPs (Marshall, 1997, p. 1753; National Advisory Council for Human Genome Research, 1997). Francis Collins, director of the National Human Genome Research Institute, suggested that ‘a public/private consortium’ might provide the means ‘to generate the data and place it in public databases’ (Collins et al., 1997; National Advisory Council for Human Genome Research, 1997). It would take almost three years of negotiations to assemble what became The SNP Consortium, but once established, it was a formidable organization, bringing together public and charitable research funders with thirteen leading pharmaceutical and information technology companies (Masood, 1999; National Human Genome Research Institute, 2000; see also Holden, 2002).^2^ Like the Merck Genome Index Project (MGIP), the SNP Consortium proved effective in generating DNA sequence data and dedicating it to the public domain. By 2005, even the most confident commercial competitors had conceded that they might as well do the same with their own SNPs (Cook-Deegan & McCormack, 2001; Marris, 2005).

The MGIP and The SNP Consortium marked a striking departure from earlier industry attitudes towards knowledge in the public domain. Through the 1960s and 1970s, pharmaceutical companies had refused to have anything to do with intellectual property over which they could not secure exclusive rights. Now they were working together with public and charitable research funders with the express purpose of creating a corpus of public-domain knowledge. It was a remarkable turnaround, indicative of the extent to which the business of genomic data production changed in the 1990s, not just in terms of technical capacity, but in terms of how new kinds of companies with new business models were disrupting the existing pharmaceutical innovation system. The decision by some of the leading pharmaceutical companies to place substantial amounts of genomic data in the public domain was essentially a defensive one, intended to preserve their privileged position by depriving biotech companies of the means to gain undue influence.

## Rethinking the utility of ESTs

At the same time as practical measures to pre-empt the patenting of certain kinds of DNA sequences were being implemented, legal experts were engaging in technical discussions about the patentability of ESTs. Some of the most searching commentary came from Rebecca Eisenberg, an academic intellectual property lawyer based at the University of Michigan. Eisenberg had begun considering DNA patents in the late 1980s (Eisenberg, 1987, 1989). At that time, she inclined to the view that such patents served the public interest. ‘Scientists would like to think that they are involved in a cooperative effort for the advancement of knowledge and the betterment of humanity’, she observed, in a review she published in 1990. ‘But this begs the question of how best to promote the advancement of knowledge and the betterment of humanity’ (Eisenberg, 1990, p. 745). While acknowledging that patenting might inhibit free sharing of scientific data, she thought it unlikely that this would seriously impede research: ‘[G]iven the substantial and growing dependence of biomedical research on private funding, we should be cautious about assuming that private intellectual property rights will retard progress in these fields’ (Eisenberg, 1990, p. 745). Such fears also needed to be weighed against the possibility that ‘the lack of intellectual property rights in DNA sequences might undermine incentives for the private sector to support subsequent research to put this information to practical use’ (Eisenberg, 1990, p. 744). On balance, Eisenberg took the view that patenting of DNA sequences best served the aims of commercial development, and hence the public good.

The outcry over NIH’s EST patent applications prompted Eisenberg to rethink. She was aware that scientists were ‘raising their voices in a now familiar refrain about the detrimental effects of patenting on scientific communications’ (Eisenberg, 1992, p. 903), but it was industry’s objections that swayed her. Organizations like the Industrial Biotechnology Association and the Pharmaceutical Manufacturers Association, she commented, ‘are not composed of naive, idealistic scientists who have limited experience with patents and limited interest in product development. Their members are the same hard-nosed, profit-maximizing firms that Congress is trying to entice into developing products from government-sponsored inventions through its patent policy’ (Eisenberg, 1993, p. 20). This was the policy, imposed on the agency by the Stephenson-Wydler Act of 1980, that NIH Director Bernardine Healy had invoked when she insisted that Venter’s EST patent applications were essential in order to ‘move closer to an understanding of how best to translate these rapidly growing numbers of gene-related discoveries into benefits for the public’ (Healy, 1992, p. 668; also Roberts, 1991b, p. 1104). But Eisenberg now questioned whether such reasoning applied to ESTs, noting that ‘views expressed to date by industry trade groups generally contradict NIH’s hypothesis that patent protection for the sequences may be necessary’ (Eisenberg, 1992, p. 907). By 1993, she was ‘at least tentatively persuaded that later product development would probably be better served by leaving the sequence information in the public domain’ (Eisenberg, 1993, p. 20)

If Eisenberg tended to favor the views of industrialists over those of scientists, her change of mind nonetheless made her a useful ally when the NIH itself decided to rethink its policy on patenting ESTs. In November 1993, the cancer biologist Harold Varmus replaced Healy as Director of NIH. One of his first actions was to convene an expert panel to advise on the EST patent applications. He included Eisenberg among the members. Three months later, Varmus announced that he was dropping the applications on the grounds that they were ‘not in the best interests of the public or science’; his decision, he added, had been ‘heavily influenced’ by Eisenberg (Anderson, 1994). Varmus went on to commission Eisenberg and another academic lawyer, Robert Merges from the Berkeley School of Law, to prepare a detailed legal opinion on the patentability of ESTs (Eisenberg & Merges, 1995).

Eisenberg and Merges considered a number of angles, but the one that would eventually prove most telling was whether ESTs met the standard of utility required for an invention to be patentable under US law. NIH’s patent applications had cited a range of potential uses for ESTs, from isolating complete genes to serving as diagnostic probes. In an initial judgement on the applications, the patent examiner had considered these claims too vague, stating that ‘the mere mention of general possible uses is not sufficient to establish a definite utility’ (Eisenberg & Merges, 1995, pp. 14–15). Eisenberg and Merges concurred: most of the specified uses, including diagnostic use, would not become practicable without further inventive activity, and as such did not constitute utility under current interpretations of patent law. On these grounds, they concluded, ‘most of the claims set forth in the NIH patent applications probably are not patentable’ (Eisenberg & Merges, 1995, p. 51). There was one important exception to this assessment, however. The NIH patent claims included use of ESTs ‘as probes to obtain full cDNA sequences and … as chromosome markers’. ‘Of all the asserted utilities for the ESTs,’ Eisenberg and Merges declared, these were ‘the most credibly operable and enabled’, given the current state of the technology (Eisenberg & Merges, 1995, p. 18), and as such, they provided the strongest grounds for claiming patentability. However, Eisenberg and Merges now questioned whether this constituted the right kind of utility to justify a patent claim. The specified uses occurred chiefly in a research context, they noted—in which case, it might be argued that they delivered ‘no *practical* benefit unless and until the full sequences themselves may be used for some purpose beyond research’ (Eisenberg & Merges, 1995, p. 18, emphasis added).

Whether or not research use constituted utility as intended in patent law, Eisenberg was well aware that distinguishing research use from other kinds of utility would not be easy in practice. Writing in 1992, she noted that ‘this is a very difficult line to draw in contemporary biotechnology research, where industrial scientists are often studying the same sorts of problems as their academic and government counterparts’ (Eisenberg, 1992, p. 905). She and Merges elaborated the point in their 1995 review of the EST claims: The distinction between research use and other forms of utility, they observed, was a ‘very difficult distinction to maintain in biotechnology in the 1990s, with researchers in government and university laboratories seeking patent protection for their discoveries and with private firms developing research tools for commercial sale’ (Eisenberg & Merges, 1995, p. 6).

In the end, they resorted to a pragmatic rather than a principled assessment of the likely consequences of patenting ESTs. On the one hand, they noted, ‘private firms are playing a growing role in generating tools for the use of genetics researchers in the public and private sectors’, and there was a risk that ‘withholding patent protection from research tools could undermine incentives to develop such tools in the private sector and to make them available to researchers’ (Eisenberg & Merges, 1995, p. 19). On the other hand, ‘there are reasons to be wary of patents on research tools, including concerns that they might be licensed on an exclusive basis to the detriment of subsequent research’ (p. 19). Faced with this dilemma, they argued, ‘it is not clear whether a strong view of the utility requirement for DNA sequences or other research tools would on balance promote subsequent research or retard it’ (pp. 19–20). In the case of ESTs, however, Eisenberg and Merges finally settled on the view that denying patent protection would probably do no harm: ‘The primary value of such sequences is in their use as research tools, a use that is unlikely to be inhibited by the absence of patent rights’ (p. 52).

Eisenberg and Merges’ cautious assessment of the patentability of ESTs ran directly contrary to the views of the US patent authorities, which at the time were taking an increasingly liberal approach to patent eligibility. Research use had long been accepted as adequate grounds for claiming utility of a wide range of products, including DNA sequences: A 1994 analysis of 1,289 human DNA sequence patents granted in the USA since 1980 found that over a third included research use among their utility claims (US Congress, Office of Technology Assessment, 1994, pp. 3–22). New PTO guidelines for patent examiners published in 1995 lowered the utility barrier further: where previous guidelines had specified that patentable inventions should possess ‘specific’, ‘credible’ and ‘substantial’ utility, the revised guidelines omitted the word ‘substantial’, effectively signalling that research use alone was sufficient to satisfy the utility requirement (US Patent and Trademark Office, 1995; Zuhn, 2001, p. 992). Two years later, the PTO explicitly declared that it considered ESTs to be patentable (Zuhn, 2001, p. 983), and in October 1998 it awarded a patent to Incyte on ESTs believed to be associated with certain pathological processes (Au-Young et al., 1998). In a letter to the journal *Science*, the PTO’s Director of Biotechnology Examination made clear that he was in no doubt that Incyte’s ESTs met the utility requirement through their potential use ‘for chromosome identification and gene mapping … [and] to identify genes that contribute to predisposition to disease’ (Doll, 1998, p. 690). Incyte’s CEO drove the point home, ‘emphatically pointing out [to reporters] that ESTs are research tools’ and comparing them to the polymerase chain reaction, ‘the use of which is also protected by patent rights and requires a license’ (Robertson, 1999).

The PTO’s actions prompted a wave of criticism, particularly from public sector scientists. Eisenberg and Merges might have struggled to demarcate research use from patentable utility, but research scientists saw no such problem. As early as 1991, the American Society of Human Genetics explained its opposition to NIH’s EST patent claims on the grounds that ‘the anticipated utility of an EST is simply that one could be used as a research tool to identify the remainder of the coding region of the gene … The EST is, at best, a starting point for further research and should not be patentable’ (American Society of Human Genetics, 1991). The committee responsible for coordinating US government-funded genome research adopted similar language in January 1992 when it declared that ESTs were ‘the “raw material” from which the discovery efforts of others will proceed’, and as such should be exempted from patent protection (NIH-DOE Subcommittee for Interagency Coordination of Human Genome Research, 1992). But the point was pressed more forcefully after the PTO announced its decision to grant Incyte’s EST patents. Varmus wrote from NIH to the patent commissioner arguing the need for strong evidence of utility in granting DNA sequence patents; while Bruce Alberts, president of the National Academy of Sciences, urged that ESTs and ‘other research tools’ should not be patented lest they hinder further research and development (Alberts, 1997; Cook-Deegan & Heaney, 2010, p. 400; Winickoff, 2013, pp. 15–21). Perhaps most persuasively, Alan Williamson invoked the Merck Gene Index Project as an indicator of industry opposition to EST patents, arguing that ‘research tools are generally “pre-competitive” for the pharmaceutical companies. The MGIP generates research tools that are of equal value to researchers in academia and the biotechnology and pharmaceutical industry.’ Williamson was adamant that ‘[s]equence information alone does not constitute a patentable invention’; whereas ‘making the EST data freely available should stimulate patentable inventions stemming from subsequent elucidation of the entire sequence, function and utility of each gene’ (Williamson, 1999, pp. 117–118).

Faced with this barrage of criticism, the PTO reviewed its position. In December 1999, responding to comments it had received ‘regarding the patentability of expressed sequence tags’, the PTO published a revised draft of its utility examination guidelines. ‘Many comments stated that sufficient patentable utility has not been shown when the sole disclosed use of an EST is to identify other nucleic acids whose utility was not known’, the document noted, while ‘several comments opined that ESTs are genomic research tools that should be available for unencumbered research to advance the public good’ (US Patent and Trademark Office, 1999, p. 71441). The revised guidelines, duly adopted in 2001, reinstated the requirement to demonstrate ‘substantial’ utility that had been dropped in 1995 (US Patent and Trademark Office, 1999; Zuhn, 2001, pp. 994–995). That guidance was subsequently affirmed by the Court of Appeal in the Federal Circuit—established in 1982 by the Reagan administration specifically to hear patent appeals, and widely seen as particularly sympathetic to the interests of industry—when in 2005 it rejected an application from Monsanto for a patent on ESTs derived from maize, on the grounds that ESTs lack utility in the absence of knowledge about the function of the gene itself (Harvard Law Review, 2006). The revised guidelines, and their interpretation by the Federal Circuit, established a threshold of utility for DNA patents which now depended, at minimum, on knowledge of biological function (Calvert, 2007).

These decisions by the PTO and Federal Circuit represented a significant if partial reversal of the expansionist approach to DNA sequence patents that had prevailed through the 1980s and 1990s. By recognizing research tools as a distinct class of things, to be excluded from the realm of patentable subject matter, the patent authorities effectively aligned themselves with the interests, not just of public-sector scientists, but also of pharmaceutical executives (see Godt, 2008). Like industry initiatives such as the MGIP and The SNP Consortium, the revised patent regulations worked to prevent biotech companies from gaining control over large tracts of genomic sequence data, and thereby greatly enhancing their power within the pharmaceutical innovation system.

## From public domain to ‘commons’

The 1990s debates over the patentability of ESTs were instrumental in shaping the idea of a ‘genomic commons’. They did so in conversation with an established line of argument about the use of unowned resources. Historical parables about the fate of the pre-modern commons—tracts of land in which medieval English communities enjoyed common rights of access and use—have featured in debates about the social and economic importance of private property since at least the 1960s. In his classic 1967 paper ‘Toward a theory of property rights’ (Demsetz, 1967), for instance, Chicago School economist Harold Demsetz argued that ‘communal property rights’ had resulted in inefficient patterns of land use. Only when that land was ‘enclosed’—brought under the control of a single owner—did it become worth investing in agricultural improvement. Legal scholar Edmund Kitch, who played a prominent role in the Chicago School assault on NIH patent policy in the early 1970s, employed a similar narrative to justify specifically intellectual property, likening patent rights to incentives created by awarding exclusive development rights to early American prospectors who found minerals on public lands (Kitch, 1977). Without such rights, Kitch argued, no-one would have invested time and money in working a mineral claim or, by extension, in developing a promising new drug candidate. Meanwhile, in a rather different sphere of debate, the ecologist, eugenicist, and ethno-nationalist Garrett Hardin was arguing that communal land ownership resulted, not in the underdevelopment of common land envisaged by Kitch and Demsetz, but in overexploitation and environmental degradation (Hardin, 1968; see also Amend, 2019; Southern Poverty Law Centre, n.d.). Published in the eminent journal *Science* under the title ‘The tragedy of the commons’, Hardin’s resonant phrase introduced the metaphor of a doomed commons to a much wider audience, far beyond the narrowly technical debates of lawyers and economists.

Hardin’s claims were roundly contested, especially by others concerned with the conservation and fair use of natural resources, including on the grounds that, contrary to what Hardin supposed, access to pre-modern commons was usually subject to legal or customary restrictions which ensured that the land was not over-exploited but used effectively (e.g., Cox, 1985). This line of reasoning was crowned in 1990 by economist Ostrom’s (1990) classic *Governing the Commons*, which argued that, given suitable forms of governance, modern-day communities should likewise be able to manage common resources in productive, sustainable and mutually beneficial ways. Ostrom’s theorization of common-pool resources would eventually win her the Nobel Memorial Prize in Economic Sciences. In the shorter term, it provided a powerful rebuttal of the idea that collective ownership was doomed to disaster, and only private property could ensure that the best use was made of available resources.

Like Hardin’s tragic dismissal before it, Ostrom’s rehabilitation of the commons quickly found its way into debates about more than just natural resource management. By the mid-1990s, it had become a regular point of reference in legal reflections on property law (C. M. Rose, 2011). Ostrom herself was initially cautious about extending her theorization of common pool resources to include non-rivalrous goods such as knowledge. But others were willing to try, including Robert Merges, who in 1996, fresh from his collaboration with Eisenberg, began exploring how Ostrom’s ideas might apply to intellectual property in general (Merges, 1996a) and scientific data in particular (Merges, 1996b).

In the event, Merges’ careful consideration of Ostromian principles was eclipsed by the work of another legal scholar that appeared in the same year. Boyle’s (1996) *Shamans, Software, and Spleens* was a passionate denunciation of the corporate enclosure of intellectual property, particularly in the fields of software and biotechnology. Boyle too portrayed this as an assault on ‘the commons’. But he conceived of the commons very differently from Ostrom and Merges. Ostrom focused on the governance of ‘common pool resources’, where ‘the members of a clearly demarked group have a legal right to exclude nonmembers of that group from using a resource’, as distinct from public domain or ‘open access regimes (res nullius) … [which] have long been considered in legal doctrine as involving no limits on who is authorized to use a resource’ (Ostrom, 2000, pp. 335–336). For Boyle, by contrast, the public domain *was* the commons. In this, he echoed the thinking of Hardin and Chicago School theorists like Kitch and Demsetz—the key difference being that where Hardin and the property theorists saw the absence of exclusionary rights as detrimental to the public good, Boyle saw it as benefiting the public by enabling the free play of creativity. Boyle would later come to acknowledge that commons might sometimes benefit from a degree of formal or informal governance (Boyle, 2013, p. 65); but his primary concern has continued to be to advocate for a public domain ‘commons of the mind’ (Boyle, 2008). Boyle’s vision of a commons free from exclusionary rights was echoed by, among others, economist and biotechnology critic Rifkin (1998) in his widely read *The Biotech Century: Harnessing the Gene and Remaking the World*.

But it was Rebecca Eisenberg who did most to introduce the idea of ‘the commons’ into mainstream policy discussions about DNA patents. As noted, she and Merges had struggled to make a principled legal case for excluding ESTs from patent protection on the grounds that they were research tools. But Eisenberg was also open to more pragmatic considerations, including that such patents could ‘add to a thicket of rights that firms must negotiate their way past before they can get their products on the market’ (Eisenberg, 1994, p. 168). An opportunity to elaborate this observation in striking and original terms arose not long afterwards, thanks to one of her colleagues in the Michigan Law faculty. Michael Heller had been researching the transition to a capitalist economy in post-communist Russia, where he noted that small retail businesses often struggled because they inherited a multiplicity of overlapping claims to ownership of different aspects of the business. Recalling Hardin’s attribution of over-exploitation to an absence of property rights, Heller argued that his own research revealed the opposite situation: a tragedy, not of the commons, but of ‘the anticommons’ (Heller, 1998). Eisenberg quickly realized that this analysis might be extended to include the contested biotechnology patents, and together she and Heller went on to publish, just four months later, a landmark paper on ‘The anticommons in biomedical research’ (Heller & Eisenberg, 1998). While ‘Hardin’s metaphor’ of the tragedy of the commons had become ‘central to debates in economics, law, and science and … a powerful justification for privatizing commons property’ (Heller & Eisenberg, 1998, p. 698), they cautioned that too many exclusionary claims on a finite resource could have a ‘mirror image’ effect. They went on to explore some of the ways that a tragedy of the anticommons could arise in the sphere of biomedical research and innovation, and ended with a warning: ‘Privatization must be more carefully deployed if it is to serve the public goals of biomedical research … Otherwise, more up-stream rights may lead paradoxically to fewer useful products for improving human health’ (Heller & Eisenberg, 1998, p. 701).

Heller and Eisenberg’s paper inserted the idea of the commons into scientific and commercial debates about intellectual property and biomedical innovation in a way that critics like Boyle and Rifkin had failed to do. Growing out of Eisenberg’s earlier reflections on the problems of patenting on ESTs and other putative research tools, her and Heller’s analysis of anticommons effects in biomedicine aligned neatly, both with the interests of scientific bodies like the NHGRI, and with the commercial sponsors of initiatives like the Merck Gene Index Project. Published, like Hardin’s classic provocation, in the journal *Science*, it reached an audience far beyond the esoteric circles of property theorists. But at the same time, in looking back directly to Hardin, Heller and Eisenberg effectively resuscitated his negative view of commons as the absence of proprietary rights. In a context of binarized debate over whether or not to patent, it was this, rather than Ostrom’s more positive vision of communally governed common pool resources, that provided the starting point for much of the talk of genomic and other biomedical commons that would follow.

## Discussion

The disputes over the patenting of certain kinds of DNA sequences that took place during the 1990s provide an object lesson in how the institutions of property, understood as ‘a relationship between people in relation to “things”’ (Macfarlane, 1998, p. 112), serve to structure social relations. The rapid development of molecular biotechnology during the 1970s and 1980s had resulted in the production of new kinds of inventions—isolated DNA sequences—that were judged to satisfy the requirements for patentability. At the same time, a new class of owners—biotech companies, and the universities and funding agencies who supported the research on which those companies were founded—was empowered to claim ownership of those products. By the early 1990s, as production of new DNA sequences accelerated rapidly, expectations of their role in diagnostics and drug discovery grew, and patent claims proliferated; it appeared increasingly likely that the biotech companies would soon occupy a controlling position in the pharmaceutical innovation system. In response, some of the larger pharmaceutical companies sought to pre-empt the biotech companies’ patent claims by generating their own DNA sequences and dedicating them to the public domain. Subsequently, under pressure from public sector scientists as well as industry, the patent authorities decided that the contested DNA sequences were ‘research tools’, and that they lacked the right kind of utility to qualify as patentable subject matter. By defining this new class of ‘things’, and excluding them from the domain of intellectual property, the patent authorities intervened to regulate the kinds of ‘relationships between people’ that were possible within the pharmaceutical innovation system. In effect, the DNA patent decisions of the 1990s and early 2000s represented an essentially conservative adjustment to one of the key ‘knowledge-control regimes’ (Hilgartner, 2017) by means of which the pharmaceutical innovation system was ordered and managed (see Sideri, 2020).

The roll-back of DNA patenting, and the influence that biotech companies were able to assert through their ownership of DNA patents, continued through the decade following the completion of the Human Genome Project, culminating in the Supreme Court’s 2013 judgement that isolated genes do not constitute patentable subject matter. In point of law, the judgement rested on the determination that isolated genes (though not cDNAs) are ‘products of nature’ (*Association for Molecular Pathology v. Myriad Genetics, Inc., 2013*). But the motivating concerns were much the same as in the case of ESTs. During the later 1990s, biotech startup Myriad had secured patents on the BRCA1 and BRCA2 genes, certain variants of which are associated with significantly elevated risk of breast and ovarian cancer. The company aggressively asserted its rights to monopolize BRCA testing, issuing cease and desist notices to clinics and researchers who continued to work with the genes. In response, a broad coalition of patient organizations, clinical service providers, and researchers combined to challenge the legality of the patents. In court, Myriad and their allies in the biotech sector rehearsed the usual arguments about the need for patents to incentivize innovation. The complainants countered that the company’s actions not only restricted patient access to care, but inhibited further innovation by preventing access to relevant clinical and genomic data (Baldwin & Cook-Deegan, 2013; Gold & Carbone, 2010; Kevles, 2012; Parthasarathy, 2017, pp. 156–171).

In the end, the Supreme Court sided with the complainants, delivering what was effectively a pragmatic decision on innovation policy. In deciding where to draw the line between a product of nature and a patentable invention, that Court argued, it was necessary to strike ‘a delicate balance between creating “incentives that lead to creation, invention, and discovery” and “imped[ing] the flow of information that might permit, indeed spur, invention”’ (*Association for Molecular Pathology v. Myriad Genetics, Inc., 2013*, p. 2116). The Court expressly adopted ‘this well-established standard’ as the appropriate criterion ‘to determine whether Myriad’s patents claim any “new and useful … composition of matter,” … or instead claim naturally occurring phenomena’ (p. 2116). By choosing the latter option, the Court decided to treat the BRCA genes as ‘basic tools of scientific and technological work’, patents on which would ‘inhibit future innovation premised upon them’ (p. 2116; see also Rai & Cook-Deegan, 2013).

Activists were quick to claim the Supreme Court decisions as ‘a major triumph for protecting genes as a commons … roll[ing] back one of the most notorious enclosures of the past generation’ (Bollier, 2013; also e.g., Briceño Moraia & Kaye, 2013). As we have seen, however, the idea that DNA sequences in the public domain constituted a genomic ‘commons’ owed much to efforts to rationalize what had started as a commercially motivated reversal of attitudes to the proliferation of biotechnology patents. As talk of the genomic commons became common currency, it also took on more of the kind of anti-corporate, pro-public coloration that critics like Boyle and Rifkin imparted to it, while the role of commercial interests in creating the commons tended to slip from view. Not everyone suffered this amnesia. Business-school-based economists of innovation had little difficulty appreciating how public domain resources like the MGIP and The SNP Consortium could serve private interests (e.g. Agrawal & Garlappi, 2007), and the same can be said of some social scientists (e.g. Cassier, 2002). But among those who prefer to represent the genomic commons as an expression of public virtue, such initiatives are usually ignored or forgotten; where they are remembered, the impulse is often to explain them away.^3^

This selective remembering is consequential in how it informs our thinking about who benefits from pharmaceutical innovation. The valorization of the commons rests heavily on an assumption that resources in the public domain naturally serve the public good. In the case of the genomic commons, insofar as that public good is explicitly articulated, it is typically equated with the delivery of new medical interventions, especially medicines. That, of course, was also the justification for the expansion of pharmaceutical property rights from the 1960s to the 1980s, and for the evolution of the commercially oriented pharmaceutical innovation system those rights enabled. The fact that, since the mid-1990s, certain limits have been set on the extent of patenting, and space has been created within that system to accommodate public-domain genomic resources, does not negate that the system as a whole still serves the same commercial interests. But calling those public domain resources a commons and framing that commons as a public good in its own right, has tended to obscure the extent to which commercial organizations, and particularly the big pharmaceutical companies, continue to be both the arbiters and the first beneficiaries of that public good.

We can draw instructive parallels with the case of generic medicines. Generics are another class of medical molecules that reside in the public domain—not, in this instance, because they are non-patentable subject matter, but because the patents on them have expired. Like the genomic commons, generic medicines come with strong connotations of public virtue. From Senator Kefauver’s efforts to undermine pharmaceutical monopolies in the 1960s (Tobbell, 2012), to the World Health Organization’s promotion of generic versions of ‘essential’ medicines since the 1970s (Greene, 2011), to the reduction of the regulatory barriers to market entry for generic drugs by the Hatch-Waxman Act of 1984 (Carpenter & Tobbell, 2011; Dutfield, 2020), patent-free competition has been repeatedly hailed as a solution to the problems of high cost and limited access to medicines, particularly for lower-income populations.

But as Greene (2014) has shown, the results of the growth of the generics sector are more mixed than such simplistic economic reasoning would suppose. Generic competition has indeed led to reductions in the price of important medicines, not least in low-income settings (e.g., Cassier & Correa, 2003)—but those reductions have not always been as great as expected, nor as uniformly distributed. In the context of what Greene (2014, p. 6) calls ‘a private sector solution to a public health problem’, commercial interests still take precedence over questions of access, and established and emergent pharmaceutical companies alike have adapted their business strategies to make the most of the growing national and international markets for off-patent and unpatented medicines. Originator companies and generic manufacturers alike find other ways of securing a measure of market exclusivity, including proprietary formulations and aggressive branding; while many generic companies, including those in middle-income countries such as India and South Africa, tend to favour high-value drugs for richer consumers over medicines for the poor. The generics market might represent ‘a new form of economic life’, as Greene (2014, p. 89) puts it, but like the genomic commons, it evolved within the existing order of market-led biomedical innovation, not in opposition to it. It may even serve to extend that order into new geographical and economic settings, as Hayden concludes from her detailed examination of the Mexican generics sector:

Calls for the expansion of the public and the commons give me pause, precisely because such expansions rhetorically and conceptually extend the normative work of the very property regimes they seek to contest. (Hayden, 2010, p. 98; see also Hayden, 2023; Peterson, 2014)

These observations align with other critiques of public domain commons, particularly as they operate internationally. In a 2004 commentary on the implications of the TRIPS agreement, legal scholars Anupam Chander and Madhavi Sunder argue that allocating goods to the public domain tends to favor those with the wealth, power and technological capacity to make use of them, while disadvantaging less privileged actors. This asymmetry, they observed, is obscured by what they call ‘the *romance* of the commons: the belief that because a resource is open to all by force of law, it will indeed be equally exploited by all’ (Chander & Sunder, 2004, p. 1332, emphasis in original). Anthropologists Coombe and Chapman (2020, p. 6) have applied this critique to medical and biotechnological research, noting that ‘relegating certain resources to “the public” not only denies claims to them, it may unevenly distribute risks, extend obligations, and enable denials of social responsibility’. Their examples, like those of Chander and Sunder, relate primarily to the Global South, where the inequalities of power and opportunity and the risk of appropriation of Indigenous knowledge and other resources in the absence of protective rights of ownership are particularly stark. But their critique is applicable wherever property regimes include a public domain—in the Global North as much as the Global South (e.g. Lezaun & Montgomery, 2015). In order to ensure that resources are not exploited in ways that further benefit the already powerful over those less advantaged, it is not enough simply to leave them in the public domain. As Prainsack has observed, ‘the very possibility to govern a resource in a fair and equitable way requires that *someone* owns it’ (Prainsack, 2019, p. 3, emphasis in original). In this case, who should own resources such as human DNA, what rights and responsibilities should that ownership entail, and how should it be governed?

Such questions echo Hilgartner’s (2009) call for more creative thinking about ‘the politics of innovation’, including who should get to decide on the direction innovation should take, and how the legitimacy of those decisions is ensured. Parthasarathy (2021, p. 20) has recently set out some of the ways in which current US policy leaves ‘crucial innovation undone, focuses attention on commodifiable solutions rather than policy or infrastructural change that are likely to yield more egalitarian benefits, and actually creates incentives for harmful innovation’. But these failings are obscured by ‘the assumption that if we simply foster the best research and development according to the priorities of scientists, engineers, and the marketplace, the benefits will simply trickle down’ (Parthasarathy, 2021, p. 20). Parthasarathy (2021) identifies the Patent and Trademark Office (PTO) as one site where a more inclusive approach to decision making could help to foster a more democratic politics of innovation (pp. 15–16). But we can go further, looking beyond the simple binary of private ownership versus the public domain, to consider how intellectual property itself might be reconstituted in ways more suited to public interests. Imagining a new ‘politics of property’, involving new kinds of ‘relationship between people in relation to “things”’ (Macfarlane, 1998, p. 112), offers another possible way of pursuing the politics of innovation.

Arguably, space is opening up for such rethinking. Ostromian ideas about common pool resources are being incorporated into the governance of ‘medical knowledge commons’, for instance (Contreras & Knoppers, 2018, p. 430; Strandburg et al., 2017)—though the political imagination informing such initiatives remains limited. Of 15 exemplary case studies discussed in a recent volume on ‘governing medical knowledge commons’, for instance, ten were comprised exclusively or largely of researchers who had come together for the purposes of sharing data. Only two were substantially led by patient support organizations, and were concerned with sharing experiential knowledge of care practices and ‘outlaw’ treatments rather than the kind of data- and technology-rich research that attracts most scientific attention and funding (Strandburg et al., 2017). For the best-resourced medical knowledge commons, in other words, the governing community is still confined to the inhabitants of the existing biomedical innovation system.

Other ways of re-ordering the politics of property and innovation are also possible, even with existing forms of intellectual property. PXE International, for instance, is run by families affected by the genetic disorder pseudoxanthoma elasticum, and manages the intellectual property rights arising from research on data and tissue provided by its members (Kanellopoulou, 2009; N. Rose & Novas, 2005; Terry et al., 2007). At the other end of the spectrum, large public-funded institutions such as biobanks might be expected to exercise stewardship on behalf of the communities they recruit as research subjects (e.g., Winickoff, 2007). Benefit-sharing initiatives, meanwhile, offer a way to distribute any goods accruing from research participation in ways that mitigate rather than reinforce inequities—an arrangement legal scholar Peter Lee describes as ‘distributive commons’ (Lee, 2009).

So far, such initiatives generally remain in thrall to the prevailing politics of commercially oriented biomedical innovation (e.g., Hayden, 2007). But they reflect a growing awareness that the institutions of property, and the purposes that property can serve, might be re-imagined, and that the politics of property might be reordered to promote a different politics of innovation. We might look, for instance, to anthropological studies of ‘possessive relations beyond the exclusive market-based rights characteristic of Western models of protection’ (Coombe & Chapman, 2020, p. 5) for ideas about how biomedical resources could be held and managed in ways that deliver common goods. Even within Western traditions, we could go much further in conceiving of new forms of commons—or new ways of ‘commoning’ (Gibson-Graham et al., 2016)—as means to the same end.

Crucially, though, any such re-imagining needs to be informed by a clear awareness of the politics of property and of innovation. Among other things, we should not let the ‘romance of the commons’ obscure the fact that commons may be constituted—as this paper shows—in ways that can serve conservative as much as progressive ends. Otherwise, as Prainsack cautions, we risk ‘the spread of a commons rhetoric that ultimately seeks to foster the interests of those who are already privileged and powerful’ (Prainsack, 2019, p. 10).

## Notes

1. The 1980 Act enabled transfer of patent rights to small businesses and non-profit organizations, but excluded large corporations on the grounds that they might obtain patents, not to develop themselves, but to block potential competitors. The Reagan administration dropped this clause in 1984 (Berman, 2008, pp. 854–855; Yi, 2015, pp. 169, 272 n.150).

2. As well as the NHGRI, The Wellcome Trust, and a number of academic research centres, the consortium included: AstraZeneca PLC, Aventis Pharma, Bayer AG, Bristol-Myers Squibb Company, F. Hoffman-La Roche, Glaxo Wellcome PLC, Novartis, Pfizer Inc., Searle (now part of Pharmacia), and SmithKline Beecham PLC, as well as Motorola, Inc., IBM, and Amersham Pharmacia Biotech.

3. The difficulty these commercial initiatives pose for commons enthusiasts is evident in the review by Contreras and Knoppers (2018). At one point, they acknowledge that The SNP Consortium was set up, ‘not out of a public interest in the free availability of data, but in order to avoid capture of valuable research tools by other private-sector firms’ (Contreras & Knoppers, 2018, p. 446). At another, they attribute the MGIP to ‘a combination of philanthropic intent and corporate self-interest’ (Contreras & Knoppers, 2018, p. 438). And at a third, they declare ‘these commons-based organizational structures’ to be ‘testament to the unusually strong shared community norms within the genomics research community, *even among researchers employed by industry*’ (Contreras & Knoppers, 2018, p. 438, emphasis added). The inadequacy of ‘norms’ to explain how scientists behave has been amply discussed elsewhere (Mitroff, 1974; Mulkay, 1976; Shapin, 2008)—including, most pertinently, Hilgartner’s (2012, 2017) detailed analysis of data sharing among public-sector genome researchers. For now, though, my point is only to demonstrate Contreras and Knoppers’s reluctance to accept the sufficiency of commercial self-interest to explain the creation of such commons.
